# Involvement of K^+^_ATP_ and Ca^2+^ channels in hydrogen sulfide-suppressed ageing of porcine oocytes

**DOI:** 10.1186/s40659-018-0187-2

**Published:** 2018-10-05

**Authors:** Jan Nevoral, Tereza Zalmanova, Kristyna Hoskova, Miriam Stiavnicka, Petr Hosek, Ales Petelak, Jaroslav Petr

**Affiliations:** 1 Biomedical Center, Faculty of Medicine in Pilsen, Charles University, alej Svobody 1655/76, Pilsen, Czech Republic; 2 Department of Histology and Embryology, Faculty of Medicine in Pilsen, Charles University, Pilsen, Czech Republic; 3 Institute of Animal Science, Uhrineves, Prague 10, Czech Republic; 4 Faculty of Science, Charles University, Prague, Czech Republic

**Keywords:** Oocyte, Gasotransmitter, Hydrogen sulfide, Ion channel, Oocyte ageing

## Abstract

**Background:**

Hydrogen sulfide has been shown to improve the quality of oocytes destined for in vitro fertilization. Although hydrogen sulfide is capable of modulating ion channel activity in somatic cells, the role of hydrogen sulfide in gametes and embryos remains unknown. Our observations confirmed the hypothesis that the K_ATP_ and L-type Ca^2+^ ion channels play roles in porcine oocyte ageing and revealed a plausible contribution of hydrogen sulfide to the modulation of ion channel activity.

**Results:**

We confirmed the benefits of the activation and suppression of the K_ATP_ and L-type Ca^2+^ ion channels, respectively, for the preservation of oocyte quality.

**Conclusions:**

Our experiments identified hydrogen sulfide as promoting the desired ion channel activity, with the capacity to protect porcine oocytes against cell death. Further experiments are needed to determine the exact mechanism of hydrogen sulfide in gametes and embryos.

## Introduction

Matured metaphase II (MII) oocytes are destined for fertilization and, therefore, represent essential cells in human reproduction, as well as assisted reproduction technologies (ART) when natural reproduction fails. However, oocyte maturation is not strictly synchronized at MII, and oocytes undergo undesirable changes related to post-ovulatory ageing. These changes ultimately manifest in cell death (i.e., apoptosis or lysis) or parthenogenetically activated embryonic development [1, 2].

Accordingly, age-related signalling has been extensively studied, and various substances with oocyte protective effects have been tested [3, 4]. Gasotransmitters, particularly hydrogen sulfide, represent potent signalling molecules involved in the regulation of oocyte maturation and ageing [3, 5, 6]. Accordingly, a hydrogen sulfide treatment suppresses the negative effects of oocyte ageing, such as parthenogenetic activation and oocyte/embryo death, in a dose-dependent manner [3]. The mechanism of hydrogen sulfide action is well studied. Indeed, hydrogen sulfide-activated ATP-sensitive K^+^ (K^+^_ATP_) ion channels have been described, while L-type Ca^2+^ ion channels have also been shown to be inhibited by hydrogen sulfide [7, 8]. S-sulfhydration, a hydrogen sulfide-derived post-translational modification [9], is considered to be the mechanism of hydrogen sulfide action towards ion channels [10]. Although the actions of hydrogen sulfide have been intensively studied in somatic cells, findings in gametes are rare [5, 11].

In the present study, we hypothesized that hydrogen sulfide also modulates the activity of K^+^_ATP_ and/or L-type Ca^2+^ ion channels in aged oocytes. We used oocytes from the well-established biomedical model of the domestic pig (*Sus scrofa*) and explored possible ways to preserve the quality of oocytes and improve their availability for ART. We have observed a protective effect of hydrogen sulfide treatment on aged oocytes and subsequently revealed hydrogen sulfide to be a signalling molecule in oocyte [reviewed by 12]. Based on known targets of hydrogen sulfide with potent cell-protective activities [13], we pharmacologically induced the activation and inhibition of K^+^_ATP_ and Ca^2+^ ion channels through minoxidil and verapamil treatment of aged oocytes, respectively. We tracked intact MII oocytes and all undesired oocyte phenotypes.

## Materials and methods

All chemicals were purchased from Sigma-Aldrich (USA) unless otherwise stated.

### Pig oocyte collection and oocyte ageing

Pig ovaries were obtained from non-cyclic gilts at a local slaughterhouse (Jatky Cesky Brod, a.s., Czech Republic) and transported to the laboratory. Cumulus-oocyte complexes were collected from 3 to 5 mm follicles by aspiration using a syringe and 20G needle. Fully grown immature oocytes with intact ooplasm and compact layers of cumulus cells were selected for in vitro maturation in modified M199 culture medium for 48 h at 39 °C and 5% CO_2_ [6]. Matured MII oocytes were denuded and subjected to further in vitro cultivation in modified M199 under standard conditions for 72 h [3].

### Pharmacological treatment of aged oocytes

During the 72 h in vitro culture of matured oocytes, minoxidil (K^+^_ATP_ channel activator), verapamil hydrochloride (L-type Ca^2+^ channel blocker) or Na_2_S·9H_2_O was added. In further experiments, Na_2_S supplementation was combined with different concentrations of glibenclamide (K^+^ channel blocker) or BAY K8644 (L-type Ca^2+^ channel agonist).

### Evaluation of oocyte ageing

**Fig. 1:**
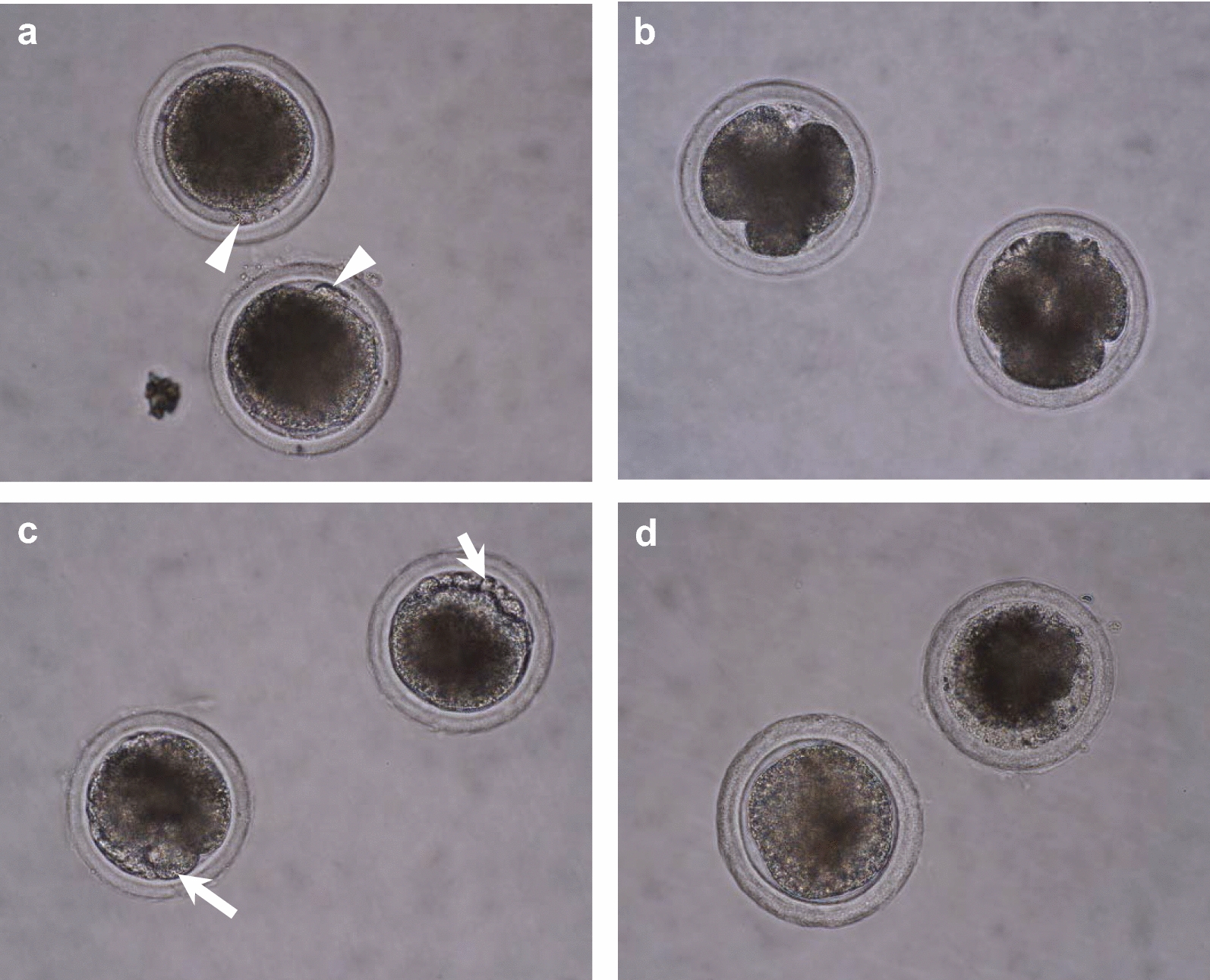
Aged porcine oocytes with different manifestations of ageing. **a** Intact MII: matured oocytes physiologically arrested in metaphase of the 2nd meiotic division. The 1st polar body is extruded (arrowhead) and marks matured oocyte destined for fertilization. **b** Activated: parthenogenetically activated oocytes with spontaneous embryonic development. **c**, **d** Cell death: oocytes underwent either fragmentation or lysis, respectively. Apoptotic bodies are indicated (arrow)

At the end of in vitro culture, aged oocytes were mounted on slides using Vaseline and fixed in acetic alcohol (1:3, v/v) for at least 48 h. Fixed oocytes were stained with 1.0% orcein and evaluated via phase contrast microscopy (Olympus, Germany). Aged oocytes were evaluated as follows: (i) intact MII oocytes without visible morphological changes; (ii) cell death, i.e. apoptosis (marked with visible apoptotic bodies, also called fragmentation) or lysis (necrosis) or (iii) parthenogenetic activation (recognized by spontaneous embryonic development). Ageing phenotypes are shown on Fig. 1.

### Statistics

Data from 120 oocytes per group in three independent experiments are expressed as the mean ± S.E.M. The data were processed using Statistica Cz 12 (StatSoft, USA). For comparisons of the study groups, one-way ANOVA (for quantitative variables) was used. In the case of a significant overall finding, differences between individual group pairs were assessed using the Bonferroni post hoc test. The level of statistical significance was set at α = 0.05.

## Results and discussion

### The modulation of ion channel activity suppresses oocyte ageing

**Fig. 2:**
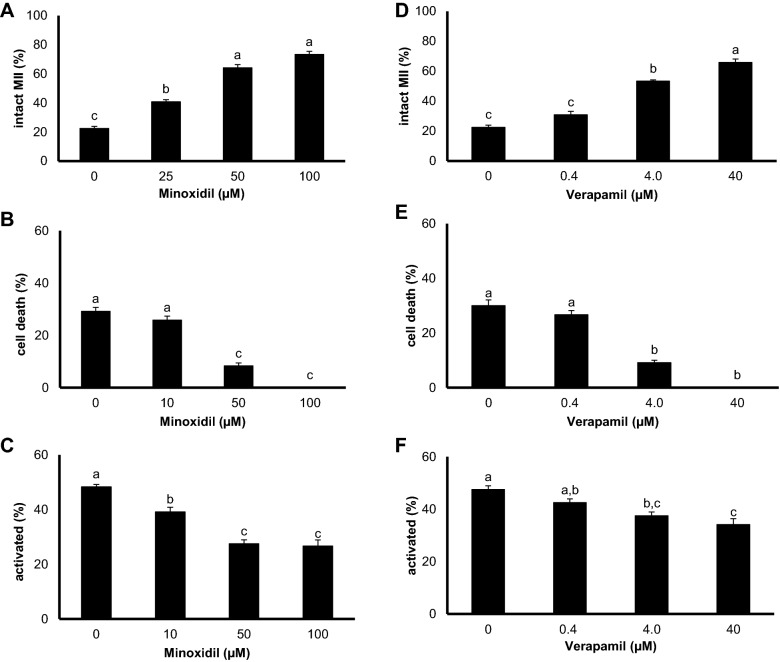
Role of ion channel modulators in oocyte ageing. **A**–**C** The protective effect of minoxidil (K^+^_ATP_ channel activator) treatment on oocyte ageing. **D**–**F** A similar effect was observed with verapamil (inhibitor Ca^2+^ channels). Specifically, **A**, **D** increased intact MII oocyte numbers, **B**, **E** suppression of apoptotic or lytic oocytes (cell death), and ultimately, **C**, **F** parthenogenetically activated oocytes. Different superscripts indicate statistically significant differences (P ≤ 0.05)

We observed an improvement in oocyte quality following the modulation of ion channel activity using the K^+^ and L-type Ca^2+^ channel activator and inhibitor, respectively. Both agents yielded a dose-dependent increases in the number of intact MII oocytes (Fig. 2A, D), along with the suppression of cell death, such as apoptosis or lysis (Fig. 2B, E). The positive effect of hydrogen sulfide on oocyte ageing [3], as well as its ability to modulate ion channel activity [reviewed by 7] have been described. Therefore, subsequent experiments were performed using combined treatment with a hydrogen sulfide donor and modulators of ion channel activity.

### K^+^ channel inhibition reduces the protective effect of hydrogen sulfide against oocyte ageing

Based on the aforementioned protective effect of hydrogen sulfide [3], we speculated that K^+^ channel activity has a positive effect on aged oocytes. Moreover, the ability of hydrogen sulfide to modulate ion channel activity is known [7, 8], as is the protective effect of K^+^_ATP_ channel activation alone (see above). Based on the ability of hydrogen sulfide to activate K^+^_ATP_ channels, we sought to reverse the positive effect of the hydrogen sulfide donor using glibenclamide, a K^+^_ATP_ channel blocker (iK_ATP_).

**Fig. 3:**
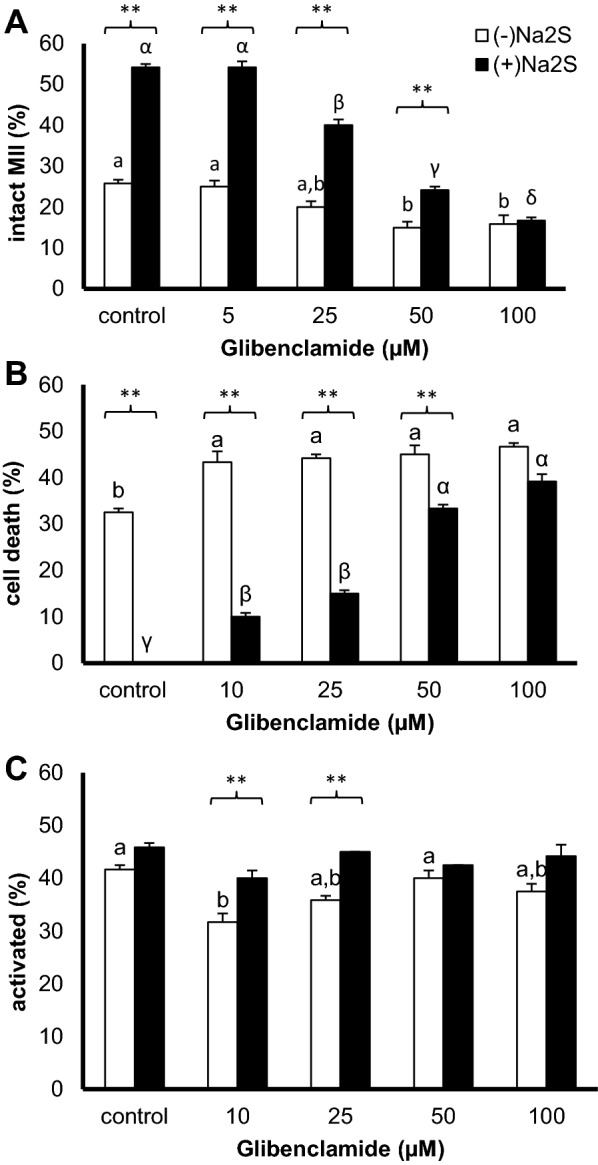
Effect of the combined treatment with a hydrogen sulfide donor and K^+^_ATP_ channel inhibitor. Na_2_S (300 µM, (+)Na_2_S) was used as the extracellular hydrogen sulfide donor, and glibenclamide (10–100 µM; iK_ATP_) was used to inhibit the K^+^_ATP_ channel both alone ((−)Na_2_S) and combined with Na_2_S ((+)Na_2_S). **A**–**C** The proportions of intact MII oocytes; cell death, including apoptosis or lysis; and parthenogenetically activated oocytes were determined, respectively. Different superscripts indicate statistically significant differences among experimental groups within a treatment (a, b; α, β, γ, δ). Asterisks indicate a significant difference between treatments within the same iK_ATP_ concentration (at α level less than 0.01)

As expected, iK_ATP_ impaired the quality of aged oocytes compared with control oocytes aged in pure medium (Fig. 3; (−)Na_2_S). In contrast, addition of a hydrogen sulfide donor alone (control oocytes for (+)Na_2_S treatment) increased the intact MII oocytes up to 54.2 ± 0.8% (Fig. 3A), while oocyte apoptosis/lysis (cell death) was completely inhibited (Fig. 3B). iK_ATP_ reduced the hydrogen sulfide-increased portion of intact MII oocytes after 72 h of oocyte ageing in a dose-dependent manner (Fig. 3A). While hydrogen sulfide-treated oocytes showed a significantly decreased prevalence of oocyte cell death (Fig. 3B), iK_ATP_ treatment reversed the positive effect of hydrogen sulfide (Fig. 3A, B). The observation is consistent with the general assumption that hydrogen sulfide acts as a K^+^_ATP_ ion channel activator, as evidenced in vascular smooth muscle cells [14], cardiomyocytes [15], neuronal cells [16] and/or pancreatic beta cells [17].

### L-type Ca^2+^ channel activation impairs the protective effect of hydrogen sulfide against oocyte ageing

In addition to K^+^_ATP_ channels, we tested the role of Ca^2+^ channels in hydrogen sulfide-protected oocytes. Consistent with our observation of the beneficial effect of Ca^2+^ channel inhibition (see above), we experimentally reversed the positive effect of the hydrogen sulfide donor using BAY K8644 an activator of L-type Ca^2+^ channels (aCa).

**Fig. 4:**
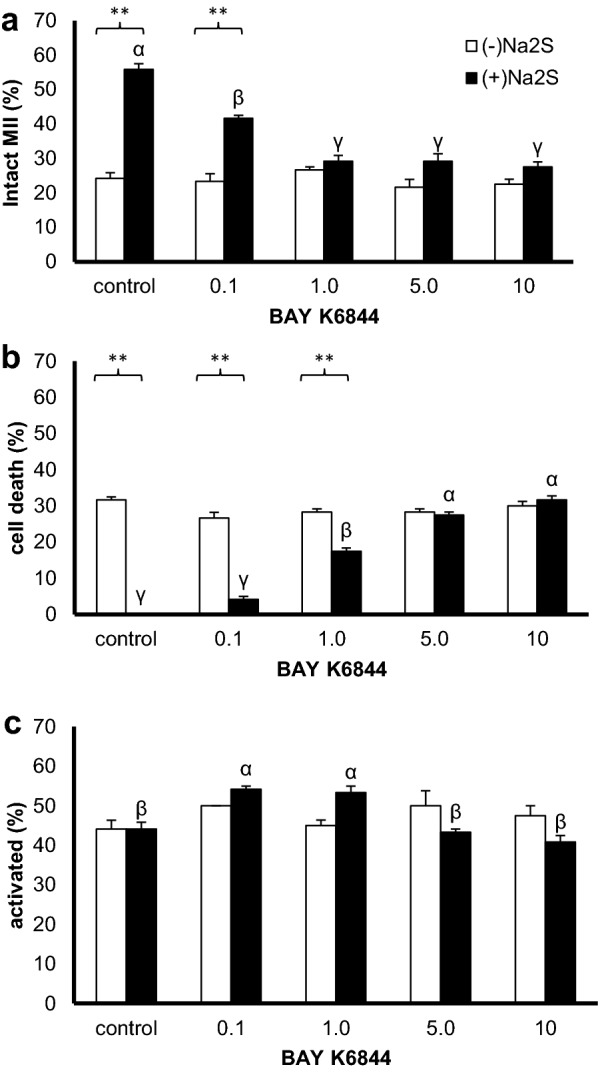
Effect of the combined treatment with a hydrogen sulfide donor and L-type Ca^2+^ channel activator. Na_2_S (300 µM, (+)Na_2_S) was used as the extracellular hydrogen sulfide donor, and BAY K6844 (0.1–10 µM; aCa) was used to activate L-type Ca^2+^ channels both alone ((−)Na_2_S) and combined with Na_2_S ((+)Na_2_S). **a**–**c** The proportions of intact MII oocytes; cell death, including apoptosis or lysis; and parthenogenetically activated oocytes were determined, respectively. Different superscripts indicate statistically significant differences among experimental groups within a treatment (α, β, γ). Asterisks indicate a significant difference between treatments within the same aCa concentration (at α level less than 0.01)

Different concentrations of the Ca^2+^ channel activator ((−)Na_2_S) had no observable effect on oocyte phenotypes (Fig. 4). When coupled with hydrogen sulfide donor treatment ((+)Na_2_S), Ca^2+^ channel activation suppressed the protective effect of hydrogen sulfide on MII oocytes (Fig. 4a). Additionally, the reduced occurrence of oocyte apoptosis or lysis (i.e., cell death, Fig. 4b) induced by hydrogen sulfide was reversed by addition of the Ca^2+^ channel activator. Our evidence suggests that hydrogen sulfide exerts is ageing-preserving effect through the suppression of Ca^2+^ channels. Our findings are in accordance with the observed intracellular Ca^2+^ elevations that accompany oocyte ageing [18]. On the other hand, the modulatory effect of hydrogen sulfide on Ca^2+^ channels is somewhat inconsistent, as hydrogen sulfide is known to activate T-type Ca^2+^ channels in neurons [19]. Therefore, the effect of hydrogen sulfide on Ca^2+^ ion channels in spermatozoon and/or embryos requires further study.

## Conclusions

**Fig. 5:**
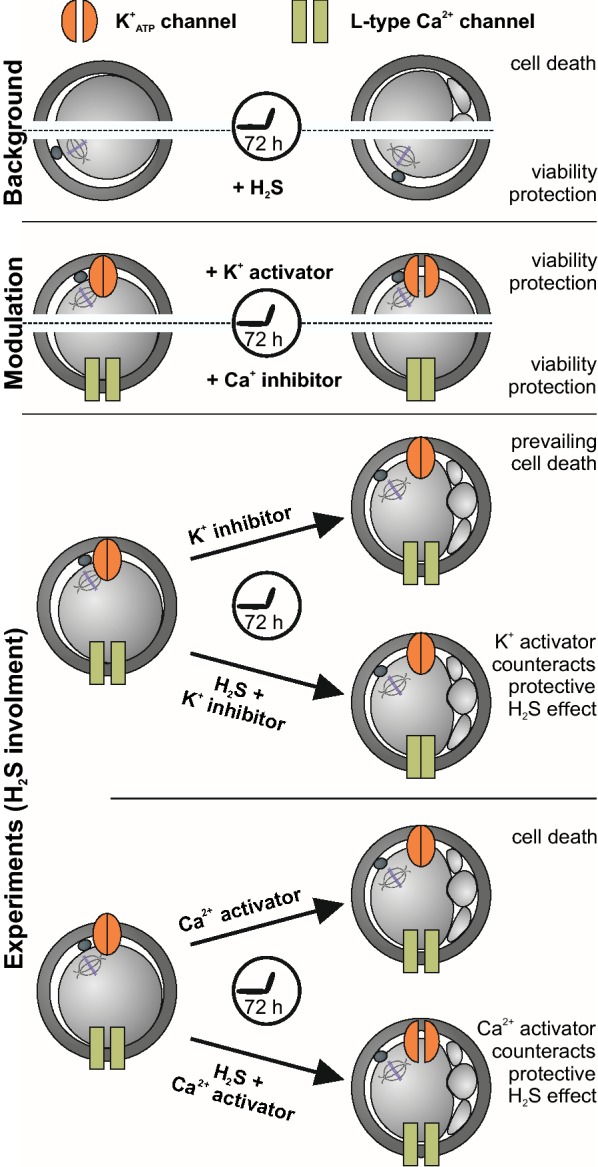
Graphical summary of oocyte ageing and the involvement of hydrogen sulfide through the modulation of ion channels. Hydrogen sulfide (H_2_S) treatment protects oocytes against cell death when fragmented or lytic oocytes are observed (background). Modulators of K^+^_ATP_ and Ca^2+^ channels (activator and inhibitor, respectively) show hydrogen sulfide-like rescue effects (modulation). Therefore, we experimentally tested the crosstalk of K^+^_ATP_/Ca^2+^ ion channels and hydrogen sulfide when the beneficial effect of hydrogen sulfide was reversed using increasing concentration of K^+^_ATP_ inhibitor or Ca^2+^ channel activator (experiments). Based on our findings, we concluded that K^+^_ATP_/Ca^2+^ channels are molecular targets of hydrogen sulfide in aged oocytes

Hydrogen sulfide supplementation represents a possible method of protecting against undesired phenotypic changes in oocytes (Fig. 5). Our observations indicate that hydrogen sulfide is able to activate the K^+^_ATP_ channel and inhibit the L-type Ca^2+^ channel. To the best of our knowledge, S-sulfhydration of cysteine thiols in proteins is a likely molecular mechanism for the effects of hydrogen sulfide in gametes and embryos. Further study and understanding of the action of hydrogen sulfide is necessary for translation to ART, which still include many undefined factors and have variable success rates.

## Abbreviations

aCa: the activator of L-type Ca^2+^ channels (BAY K8644)
ART: assisted reproductive technology
iK_ATP_: the K^+^_ATP_ channel blocker (glibenclamide)
H_2_S: hydrogen sulfide
K^+^_ATP_: ATP-sensitive K^+^ ion channels
MII: metaphase II (2nd meiotic division)
Na_2_S: Na_2_S·9H_2_O, sodium sulfide nonahydrate
